# Computational studies of flaviviruses: approaching to novel fusion inhibitors

**DOI:** 10.1186/1758-2946-4-S1-P29

**Published:** 2012-05-01

**Authors:** Dmitry I Osolodkin, Liubov I Kozlovskaya, Galina G Karganova, Evgenia V Dueva, Vladimir A Palyulin, Nikolay S Zefirov, Vladimir M Pentkovski

**Affiliations:** 1 Department of Chemistry, Moscow State University, Moscow 119991, Russia; 2 Chumakov Institute of Poliomyelitis and Viral Encephalitides RAMS, Moscow region 142782, Russia; 3 Moscow Institute of Physics and Technology, Dolgoprundny 141700, Russia

## 

Flaviviral infections affect millions of people throughout the world, in such different territories as Europe, Australia, USA, and China. Among these infections are dengue fever, tick-borne encephalitis, Powassan encephalitis, West Nile fever, yellow fever, Omsk haemorrhagic fever, etc. Effective vaccines exist against only several flaviviruses. Moreover, there is no other specific therapy for flaviviral infections; consequently, new antiviral drugs are needed for the treatment of these diseases.

Falviviruses are characterised by enveloped virion with E protein forming its outer surface. During the entry to the target cell E protein undergoes pH-induced conformational switch into fusogenic state that drives fusion of virion and cell membranes with consequent release of the viral genome into cytoplasm.

Our studies are devoted to the modelling of flavivirus virion envelope proteins structure and molecular dynamics simulation, generally aiming in design of the fusion inhibitors. First, we studied ectodomain of TBEV E protein and revealed the possible mechanism of differences in virus binding to glycosaminoglycans [1]. Second, the analysis of point substitutions in TBEV E protein ectodomain in MD simulation study revealed the basis of their influence on the virion properties. These studies were expanded to include the stem and anchor region of this protein and describe its interactions with the viral membrane. The global aim of this study is an atom-scale simulation of viral fusion process.

Another aspect of the fusion inhibitor design is analysis of the inhibitor binding site composition and structure-based virtual screening of the putative inhibitors. Such analysis has been performed for DENV, TBEV and POWV and allowed identifying molecules with high probability to inhibit the crucial step of these infections.
